# Changes of CB1 Receptor Expression in Tissues of Cocaine-Exposed Eels

**DOI:** 10.3390/ani15121734

**Published:** 2025-06-12

**Authors:** Lorenzo Riccio, Teresa Chianese, Aldo Mileo, Sabrina Balsamo, Rosaria Sciarrillo, Roberta Gatta, Luigi Rosati, Maria De Falco, Anna Capaldo

**Affiliations:** 1 Department of Veterinary Medicine and Animal Production, University of Naples Federico II, Via Federico Delpino, 80137 Naples, Italy; lorenzo.riccio@unina.it; 2 Department of Biology, University of Naples Federico II, Via Cinthia, Edificio 7, 80126 Naples, Italy; teresa.chianese2@unina.it (T.C.); aldo.mileo@unina.it (A.M.); sab.balsamo@gmail.com (S.B.); madefalco@unina.it (M.D.F.); anna.capaldo@unina.it (A.C.); 3 Department of Science and Technologies, University of Sannio, Via F. de Sanctis snc, 82100 Benevento, Italy; sciarrillo@unisannio.it; 4 OncoSwab Co., 166 Geary St, 15th Floor, Suite 33, San Francisco, CA 94108, USA; roberta.gatta@oncoswab.com; 5 National Institute of Biostructures and Biosystems (INBB), 00136 Rome, Italy; 6 CIRAM-Interdepartmental Research Centre “Environment”, University of Naples Federico II, Via Tarsia 31, 80134 Naples, Italy

**Keywords:** *Anguilla anguilla*, cannabinoid receptor 1, environmental cocaine, gills, gut, kidney, liver, muscle, ovary, skin

## Abstract

Previous studies performed on the European eel *Anguilla anguilla* showed changes in the morphology and physiology of several tissues after exposure to environmental cocaine concentrations. To better understand the model through which cocaine produced its effects on these tissues, we investigated whether there were alterations in the expression of cannabinoid CB1 receptor (CB1R). Indeed, the endocannabinoid system, and CB1R, regulate neurotransmission, neurodevelopment, embryonic development, reproduction, and the activity of the gastrointestinal system. CB1R has been detected in nervous and peripheral tissues in mammals, and orthologues of the mammalian CB1R are found throughout vertebrates including chicken, turtle, frog, and fish. Therefore, samples of gut, kidney, ovary, muscle, liver, skin, and gills from cocaine-exposed and non-exposed eels were processed for routine histology. Immunohistochemical analysis was carried out to evaluate the immunolocalization of the CB1R. Our results showed for the first time (1) the presence of CB1R in the peripheral tissues of the eel, and (2) statistically significant differences in the localization of CB1R in the gut, kidney, ovary, muscle, and liver of the eels exposed to cocaine, compared to controls. These results demonstrate the involvement of CB1R in cocaine effects and suggest its potential role as a biomarker of tissue alteration.

## Simple Summary

Cocaine is, along with other illicit drugs, a contaminant of the environment, and mainly of fresh and marine waters, into which it flows after incomplete elimination by treatment plants. In contaminated waters, cocaine acts on living organisms like eels, which live for a long time in the same aquatic environment. Previous studies showed alterations in the peripheral tissues of eels exposed to cocaine; to better understand the process behind these alterations, we tested whether cocaine was able to affect the endocannabinoid system, an endogenous system playing a key role in many physiological activities of the body, and, in particular, if it was able to modify the expression of the cannabinoid receptor type 1, CB1. Samples of gut, kidney, ovary, muscle, liver, skin, and gills from cocaine-exposed and non-exposed eels were subjected to immunohistochemical analysis for CB1. Results showed for the first time (1) the presence of CB1 in the peripheral tissues of the eel, and (2) statistically significant differences in the presence of CB1 in the gut, kidney, ovary, muscle, and liver of eels exposed and not exposed to cocaine. These results demonstrate receptor involvement in the response to cocaine and suggest its possible role as a biomarker of tissue alterations.

## 1. Introduction

The results of numerous studies have shown that the presence of illicit drugs and their metabolites in both fresh and coastal water is a widespread problem [1,2,3,4,5,6], which has its origin in the growing consumption of illicit substances by the human population [7] and, in many cases, in the lack of effective removal of these substances by wastewater treatment plants [8,9,10]. As a result, the aquatic organisms, if living in contaminated waters, are exposed to continuous contact with these drugs and their metabolites [11,12,13,14,15]. The scientific literature reports many bioaccumulation, genetic, metabolic, systemic, nervous, and endocrine effects observed in the exposed organisms due to the remarkable pharmacological properties exhibited both by illicit drugs and their metabolites [5,12,13,14,16]. Previous studies on the European eel have shown that chronic exposure to low environmental concentrations of cocaine, one of the most common illicit drugs found in aquatic environments, led to its accumulation in the brain and peripheral tissues of exposed specimens, as well as alterations of organs such as liver, kidney, intestine, gills, muscle, and skin, and also affecting the endocrine system [14]. To better understand the mechanism of action of cocaine, we decided to test whether it can induce changes in the expression of the CB1 cannabinoid receptor (CB1R) in altered tissues compared to controls; in fact, given the multiplicity of actions of the CB1 receptor and its involvement in the main physiological activities of the organism, we expected a variation in response to the administration of cocaine. CB1R belongs to the endocannabinoid system (eCB), a widely preserved signaling system in vertebrates and many invertebrates, whose main function is to maintain homeostasis [17,18,19,20]. eCB consists of the endocannabinoids themselves, the enzymes involved in their synthesis and degradation, and the cannabinoid receptors. The best known and studied endocannabinoids are N-arachidonoyl-ethanolamine (AEA; anandamide) and 2-arachidonoylglycerol (2-AG), produced, respectively, by N-acyl-phosphatidylethanolamine-specific phospholipase D (NAPE-PLD) and diacylglycerol lipase (DAGL) [17,18,19,20]. The main but not only enzymes involved in the degradation of endocannabinoids are fatty acid amide hydrolase (FAAH) and monoacylglycerol lipase (MAGL) [21,22]. Finally, the main cannabinoid receptors, often present in different isoforms and subtypes in different organisms, are CB1R and the CB2 cannabinoid receptor (CB2R), belonging to the large family of seven transmembrane-spanning G-protein-coupled receptors (GPCRs) [23,24,25]. However, both AEA and 2-AG may also interact with different receptors such as the transient receptor potential cation channel subfamily V member 1 (TRPV1), peroxisome proliferator-activated receptor alpha receptors, and the orphan G-protein coupled receptors, GPR55 and GPR119 [26,27].

The choice to investigate whether cocaine effects may at least partially involve changes in CB1R expression was justified by (1) the widespread presence of CB1R throughout the animal kingdom, in peripheral tissues, as well as in the nervous system, and (2) CB1R involvement in the response to stress and addiction as well as in the main physiological activities of the organism [17,18,19,20]. CB1R is a heptameric membrane receptor, coupled to G proteins. The CB1-activated signal transduction pathway includes both the inhibition of adenylate cyclase through the action of protein Gi and the consequent activation/inhibition of Ca^2+^ and K^+^ channels, and the activation of mitogen-activated kinases [23,28,29]. The G proteins associated with these receptors are of type Gi/o; the subunit Gα inhibits cAMP production, resulting in decreased phosphorylation of protein kinase A (PKA), involved in cellular communication pathways such as the regulation of ion channels and FAK kinases [23,28,29]. CB1R may also be associated with the Gs protein, thus increasing cAMP levels, although the physiological significance of this duality has yet to be investigated [23,24,25]. CB1R bound to the Gq/11 protein activates phospholipase C (PLC) and constitutively activates nitric oxide synthase cNOS, and also mediates the release of Ca^2+^ from intracellular deposits resulting in increased concentration of intracellular Ca^2+^. Finally, CB1R positively regulates the pathway of MAP (mitosis activating protein) kinases [23,28,29]. CB1R is involved in many activities such as neurotransmission, neuro- and embryonic development, reproduction, food intake, thermoregulation, energy balance, metabolism, stress reduction, and motivation, among others [18,30]. Moreover, several studies suggest its involvement in drug addiction in general, particularly in the effects of cocaine [31,32,33,34,35]. In addition to the nervous system, CB1R has also been found in adipocytes, endothelial cells, hepatocytes, in skeletal and smooth muscle, skin, bone, in the cardiovascular system, the gastrointestinal tract, the enteric nervous system, the reproductive system, and different types of cancer [20,22]. In mammals, CB1R has been detected in nervous and peripheral tissues, and orthologues of the mammalian CB1R are found throughout vertebrates including chicken, turtle, frog, and fish [36]. In teleost, a likely gene duplication event in a common ancestor followed by retention and/or loss of duplicate genes has in many cases resulted in copies of CBIR and CB2R. In zebrafish, there is only one CB1 gene and two CB2 genes, while in *Fugu rubripes*, two CB1-type genes (CB1A and CB1B) and a single CB2 gene have been identified. In *Solea solea*, two forms of CB1R have also been found, CB1A and CB1B, whose deduced amino acid sequences are 80% identical, whereas only one CB1 receptor type was identified in *Pelvicachromis pulcher*, *Carassius auratus*, and *Sparus aurata* [17]. In the European eel, the existence of a cannabinoid receptor of 469 aa has been inferred from homology [37] (UniProt: A0A9D3MIM1_ANGAN). Moreover, a cannabinoid receptor 2 gene (cnr2), located on chromosome 1, has been identified (Bethesda, National Library of Medicine: https://www.ncbi.nlm.nih.gov/gene/118235455/#general-protein-info, accessed on 8 June 2025). To explore the mechanism underlying histological changes in the tissues of cocaine-exposed eels, the presence and distribution of CB1R was investigated by an immunohistochemical analysis and a semiquantitative analysis of the immunoreactivity.

## 2. Materials and Methods

### 2.1. Animals

In the study, we included female specimens of the European eel (*A. anguilla*), in silver stage, with the following characteristics: 52.0 ± 3.93 cm and 290.70 ± 36.40 g, mean ± s.d. The animals were purchased from a fish dealer and acclimatized in our laboratory for one month in 300 L glass aquaria under natural photoperiod. The water in the aquaria was dechlorinated and well-aerated, with the following characteristics: dissolved oxygen 8.0 ± 0.7 mg L^−1^, ammonia < 0.1 mg L^−1^, salinity 1‰, pH 7.4 ± 0.8, and temperature 15 ± 1 °C. The water in all the aquaria was replaced with fresh tap water every 24 h; furthermore, since silver stage eels do not eat, they were not fed. This study was carried out in accordance with EU Directive 2010/63/EU for animal experimentation and institutional guidelines for care and use of laboratory animals and was authorized by General Direction of Animal Health and Veterinary Drugs of the Italian Ministry of Health (Authorization n.221/2015-PR and Authorization n. 22/2015-PR).

### 2.2. Experimental Design

A stock solution of 0.006 mg mL^−1^ free base cocaine (≥97% purity, Sigma-Aldrich Inc., St. Louis, MO, USA) in ethanol was prepared. In this study a total of twenty-seven eels were used; the number of animals was decided based on the minimum number of animals from which significant results could be obtained (the three R criterion). The animals were randomly divided into three groups (eels exposed to cocaine, eels exposed to ethanol at the same concentration of the cocaine-exposed specimens, and eels exposed to tap water only) of 9 animals each. A block system was chosen for randomization, in which the animals were initially divided into groups, each containing similar animals in weight and length. Within each group, the animals were subsequently randomized to the three different experimental groups. The experiment was carried out in triplicate; therefore, the specimens in each experimental group were divided into three aquaria containing 3 specimens each one. Three control groups of three eels each were dipped in tap water only, while three groups of three eels were exposed to a nominal dose of 20 ng L^−1^ cocaine (1 mL of stock solution, added to the aquaria after water changes every 24 h) and three control groups of three eels each were exposed to ethanol. Water removed from the aquaria containing experimental groups was stored in special containers for three days before being discharged as wastewater, since nearly 90% of cocaine is degraded in 24 h at room temperature in water [38]. All groups were kept in 300 L glass aquaria under the conditions described above, and water was changed every 24 h; the exposure lasted 30 days. The eels were checked daily throughout the experimental treatment for signs of disease/stress; however, they appeared to be in good condition and were included in the evaluation of results. Each aquarium was numbered and identified by a label showing the dates and time on which measurements were made, and the values of the measurements taken; the association between the number and the experimental group was reported in a separate notebook. Water change, measurements, and administration of cocaine or ethanol took place between h.9 and h.14, with an order randomized daily, so each experimental group was tested at a different time each day. After the exposure, eels were anesthetized using MS-222 (ethyl 3-aminobenzoate, methanesulfonic acid salt 98%, Aldrich Chemical Corporation Inc., Milwaukee, WI, USA) at a concentration of 100 mg L^−1^, buffered with sodium bicarbonate at neutral pH. The specimens were weighed, measured, and killed by decapitation (Authorization n.221/2015-PR and Authorization n. 22/2015-PR). Gut, skeletal muscle, kidney, liver, ovaries, dorsal skin, and gills were sampled from each animal and processed for light microscopy. After decapitation, the tissues were immediately fixed in Bouin’s solution for 24 h, dehydrated in graded alcohols, cleared in Histolemon, and embedded in Paraplast. Tissue samples from each animal (*n* = 27) were sectioned with a microtome (Rotary 3003, pfm, BioOptica, Milan, Italy) into 4 μm thick sections, placed on poly-L-lysine slides (Menzel-Glaser, Braunschweig, Germany), and stained with a routine histological staining to verify the morphology of tissues.

### 2.3. Western Blot

Since no eel-specific CB1R has been characterized, we used Western blot to validate our antibody to CB1R. The eel kidney was potter homogenized at 4 °C with 5 mL of RIPA buffer containing 50 mM Tris-HCl (pH 7.4), 150 mM NaCl, 1 mM EDTA, 10% NP-40, and protease inhibitor cocktail (3 mM aprotinin, 1 mM leupeptin, 1 mM e-64, 130 μM bestain, 1 mM EDTA, and 2 mm AEBSF) [39]. The homogenate was centrifuged at 7000× *g* for 10 min at 4 °C. Proteins were quantified using the PIERCE method. This type of analysis was carried out with the aim of validating the anti-CB1R (SAB4500345, Sigma-Aldrich). For this purpose, 40 μg of protein from each sample was loaded, after boiling for 5 min in SDS buffer [50 mM Tris-HCl (pH 6.8), 2 g 100 mL^−1^ SDS, 10% (*v*/*v*) glycerol, 0.1 g 100 mL^−1^ Bromophenol blue], run on 12.5% SDS/polyacrylamide gels, and transferred onto a nitrocellulose membrane using a Mini trans-blot apparatus (Bio-Rad Laboratories, Hercules, CA, USA). The membrane was blocked at room temperature, then blocked with 7% non-fat milk dissolved in TBS 1×- 0.025% Tween20 for 45 min and then with 7% BSA in TBS 1×-Tween20 for a further 45 min. The membranes were incubated overnight at 4 °C with anti-rabbit CB1R and diluted 1:500 in TBS-T 2.5% BSA. Then, the membranes were washed three times with TBS-T and incubated for 1 h with anti-rabbit IgG secondary antibody (Santa Cruz, Cat. Sc-2005) diluted 1:2000 in TBS-T and 2.5% BSA. The results were acquired with Chemidoc Biorad.

### 2.4. Immunohistochemical Analysis

Slides from 3 eels per each experimental group (*n* = 9) were first deparaffined in xylene and then rehydrated using a graded series of alcoholic solutions. For antigen unmasking, slides were heat-treated in the microwave for 20 min in 10 mM citrate buffer (pH 6.0). Further, slides were incubated with 2.5% hydrogen peroxide (H_2_O_2_) for 30 min to inhibit endogenous peroxidase activity, and non-specific blockade was performed by incubation for 1 h at room temperature with normal goat serum (Pierce, Rockford, IL, USA). Sections were then incubated at 4 °C overnight with the following primary antibody diluted in normal goat serum: rabbit anti-CB1R (1:250, SAB4500345, Sigma-Aldrich). The following day, slides were washed two times (5 min each) in PBS, and a 1-h incubation at room temperature with HRP-conjugated goat anti-rabbit secondary antibody diluted 1:200 in normal goat serum was performed. The immunoreactivity signal was detected using diaminobenzidine (DAB) as chromogen, followed by counterstaining with Carazzi’s haematoxylin [40]. For negative controls, the primary antibody was omitted during incubation. The images were acquired using an Axiocam MRc5 camera (Carl Zeiss, Oberkochen, Germany) and Axiovision 4.7 software (Carl Zeiss) [41].

### 2.5. Semiquantitative Analysis of the Immunoreactivity

The immunoreactivity of the antibody was evaluated, and its cellular localization was reported. A semiquantitative assessment of all immunohistochemical stained sections was performed on 5 randomly selected slides for each tissue. For each slide, twenty randomly chosen not-overlapping fields at high power magnification (40×) were photographed under a Zeiss Axioskop microscope coupled to a video camera (Axiocam MRc5) and Axiovision 4.7 software. Each photo was elaborated with ImageJ (version 1.54): a color deconvolution filter (H DAB) was applicated to each photo to discriminate browns and blues; the obtained 8-bit browns level was binarized using a threshold manually adjusted (between 0 and 100, and 0 and 120), as needed, for each image to reflect chromogen distribution in the regions of interest [42,43]. The overall score of immunopositive cells was calculated by averaging scores of all examined cases of the same treatment group (three tissue per treatment), while the relative percentages of immunopositive cells were expressed as mean ± standard deviation (SD). The immunolabeling grade was scored as follows: 0 (Absent), 1 (Faint < 10%), 2 (Moderate 10–30%), and 3 (Strong > 30%).

### 2.6. Statistical Analysis

The member of the research team that administered the different substances was the only one aware of who was in the different experimental groups throughout the study, from experimental processing to data analysis. Statistical analysis was performed using GraphPad (version 10.4.1; GraphPad Software Inc., San Diego, CA, USA). Differences among each histological semiquantitative score were evaluated using two nonparametric tests: the Mann–Whitney test and Kruskal–Wallis test followed by a post hoc multiple comparison using Dunn’s test. The nonparametric tests were used to assess differences in the number of CB1R-positive cells evaluated between control and exposed groups; *p*-values < 0.05 were considered statistically significant.

## 3. Results

Since no differences were found between the specimens exposed to water and to the vehicle (ethanol), the results related to the vehicle-exposed specimens have not been shown.

### 3.1. Western Blot

**Figure 1:**
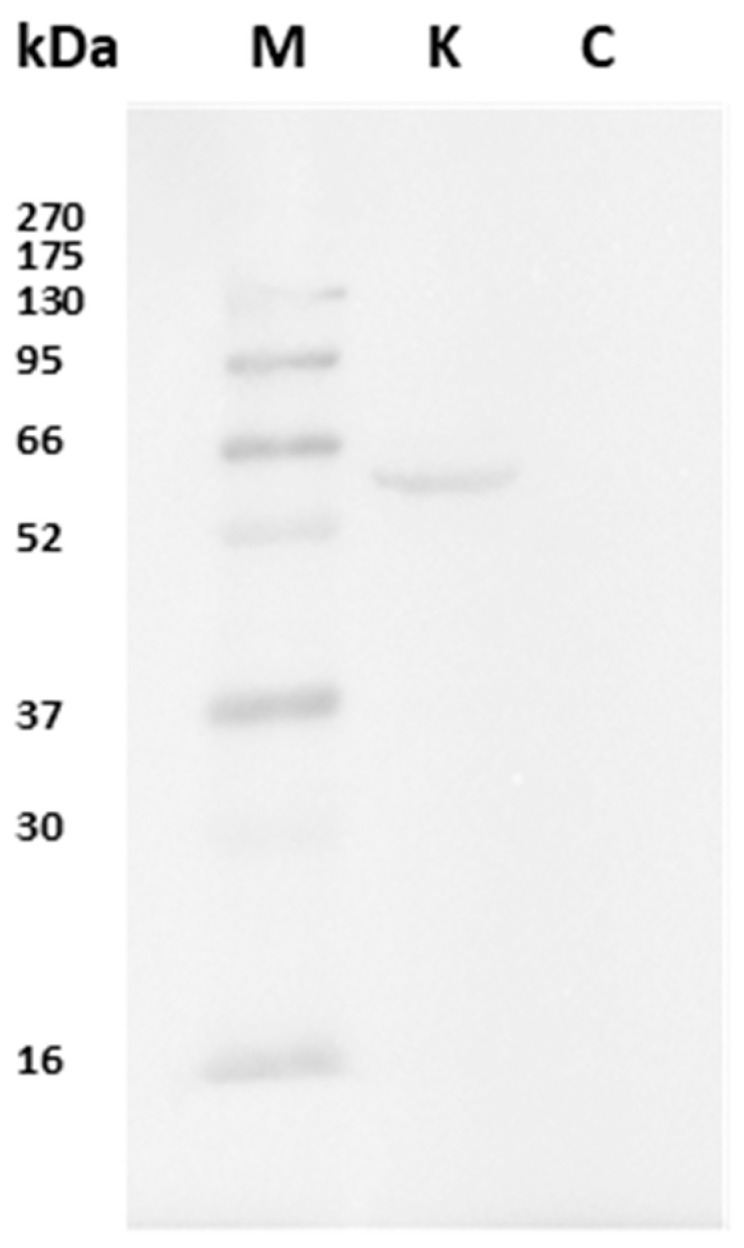
Immunoblot on eel kidney proteins. The antibody reacts with a band of about 52 kDa, corresponding to the molecular weight of CB1R. M: known molecular weight protein markers; K: kidney proteins; C: negative control shows no band.

As regards Western blot, the CB1R antibody detected a single molecular band of approximately 52 kDa (Figure 1), corresponding to the expected molecular weight of the CB1 protein, confirming the specificity of antibody binding. The absence of signals under negative control conditions, obtained by omitting the primary antibody, excludes the possibility of non-specific reactions or interference by the secondary antibody (Figure 1).

### 3.2. Gut and Liver Immunohistochemistry

**Figure 2:**
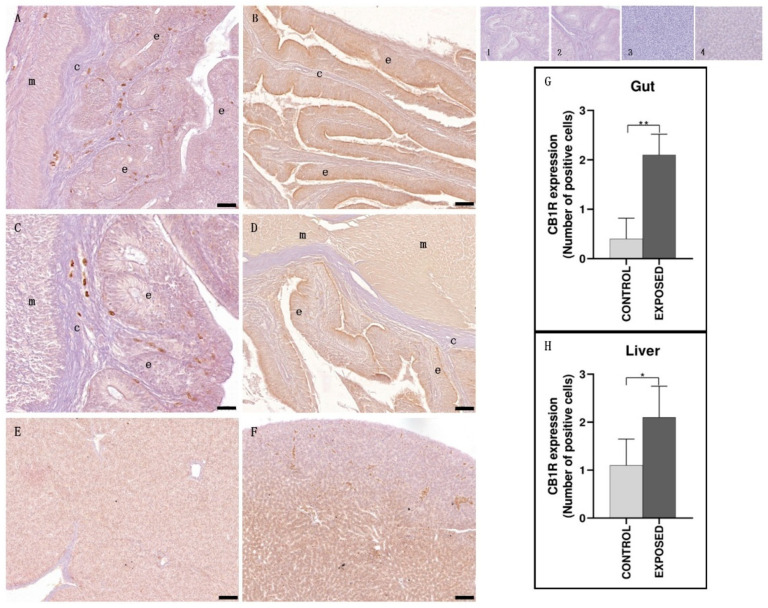
Light micrographs of eel gut and liver. Immunoreactivity to CB1R. (**A**,**C**,**E**): control specimens; (**B**,**D**,**F**): exposed specimens. Insets: 1, 2, 3, 4: negative controls of (**A**,**B**,**E**,**F**), respectively. In the gut of control specimens (**A**,**C**), the immunoreactivity was almost absent, both in epithelium (e) and in smooth muscle (m) tissue. In the exposed specimens (**B**,**D**), the smooth muscle (m) tissue and the epithelium (e) appeared immunopositive. c; connective tissue. The hepatic parenchyma of cocaine-exposed specimens (**F**) showed an increased immunopositivity to CB1R compared with control specimens (**E**). Scale bar: (**A**,**B**,**E**): 0.050 mm; (**C**,**D**): 0.020 mm; (**F**): 0.100 mm. (**G**,**H**): semi-quantitative analysis of gut and liver immunoreactivity, respectively. The Mann–Whitney test showed a statistically significant difference among the control and the exposed specimens. ** *p* < 0.01 (0.079); * *p* < 0.05 (0.0397).

Both in the gut and liver, CB1R distribution was similar. In control specimens, gut epithelium and smooth muscle tissue showed an absent-to-faint immunopositivity, whereas in cocaine-exposed specimens, both tissues showed a moderate-to-strong immunopositivity (Figure 2). In the same way, in the liver of control specimens, the immunopositivity appeared poorly distributed, whereas in cocaine-exposed specimens, a greater immunopositivity was found (Figure 2). These results were confirmed by statistical analysis that showed statistically significant differences in the localization of CB1R among the control and the exposed specimens (*p* < 0.05) (Figure 2).

### 3.3. Kidney Immunohistochemistry

**Figure 3:**
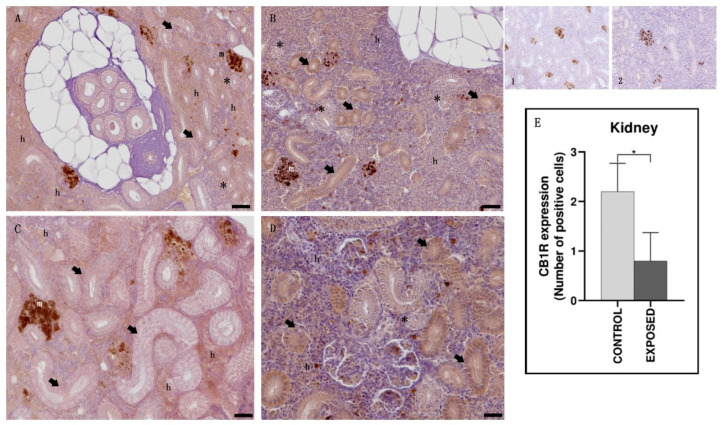
Light micrographs of eel kidney. Immunoreactivity to CB1R. (**A**,**C**): control specimens; (**B**,**D**): exposed specimens. Insets: 1, 2: negative control of (**A**,**B**), respectively. In the control specimens (**A**,**C**), the hematopoietic (h) tissue appeared immunopositive to CB1R, while the proximal (arrow) and the distal (asterisk) tubules) were not. In the exposed specimens (**B**,**D**), the immunopositivity of the hematopoietic (h) tissue appeared absent/faint, while the proximal (arrow) tubules were generally immunopositive, and the distal (asterisk) tubules were generally not. m: melanomacrophage centers. Scale bar: (**A**,**B**): 0.050 mm; (**C**,**D**): 0.020 mm. (**E**): semi-quantitative analysis of immunoreactivity. The Mann–Whitney test showed a statistically significant difference among the control and the exposed specimens. * *p* < 0.05 (0.0159).

In control specimens, the hematopoietic tissue showed a moderate-to-strong CB1R immunopositivity, whereas both the proximal and the distal tubules appeared generally immunonegative. In the exposed specimens, the immunopositivity of the hematopoietic tissue appeared absent/faint, whereas a mild-to-moderate immunopositivity to CB1R was found in proximal tubules. The distal tubules appeared generally immunonegative (Figure 3). Statistical analysis showed statistically significant differences in the localization of CB-1R among the control and the exposed specimens (*p* < 0.05) (Figure 3).

### 3.4. Ovary Immunohistochemistry

**Figure 4:**
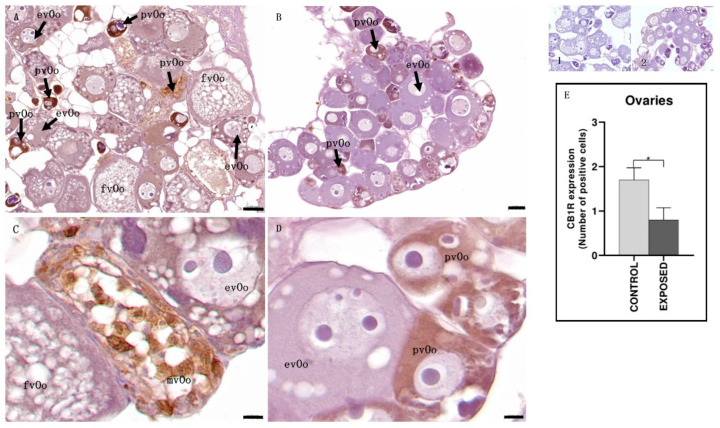
Light micrographs of eel ovary. Immunoreactivity to CB1R. (**A**,**C**): control specimens; (**B**,**D**): exposed specimens. Insets: 1, 2: negative control of (**A**,**B**), respectively. In control specimens (**A**,**C**), CB1R signaling was mainly observed in previtellogenic (pvOo) oocytes and mid-vitellogenic oocytes (mvOo), with no immunopositivity detected in early vitellogenesis (evOo) or full vitellogenesis (fvOo) oocytes. In contrast, the exposed specimens (**B**,**D**) showed immunoreactivity to CB1R only in previtellogenic oocytes (pvOo), while early vitellogenic oocytes (evOo) remained negative. Scale bar: (**A**,**B**): 0.050 mm; (**C**,**D**): 0.010 mm. (**E**): semi-quantitative analysis of immunoreactivity. The Mann–Whitney test showed a statistically significant difference among the control and the exposed specimens. * *p* < 0.05 (0.0286).

Histological analysis revealed an asynchronous ovarian development in the exposed animals compared to controls. Specifically, the ovaries of the control animals were mainly composed of fully vitellogenic oocytes, that is, large oocytes that had completed vitellogenesis. In contrast, the ovaries of cocaine-exposed animals exhibited an altered oocyte developmental progression, characterized by the presence of oocytes at early growth stages, including previtellogenic oocytes, which lacked yolk vesicles, and early vitellogenic oocytes, which showed partial yolk vesicle accumulation in the cytoplasm and a centrally located nucleus. Immunohistochemical analysis revealed a differential distribution of the CB1R. As a result of this asynchronous development determined by cocaine, in control specimens, CB1R was mainly expressed in previtellogenic oocytes and in mid-vitellogenic oocytes (Figure 4), whereas, in cocaine-exposed specimens, only previtellogenic oocytes showed a strong positivity to CB1R (Figure 4). Statistical analysis showed statistically significant differences in the localization of CB1R among the control and the exposed specimens (*p* < 0.05) (Figure 4).

### 3.5. Skeletal Muscle Immunohistochemistry

**Figure 5:**
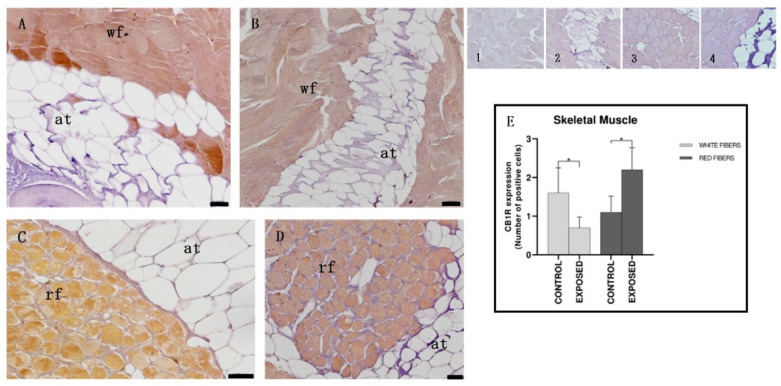
Light micrographs of eel skeletal muscle. Immunoreactivity to CB1R. (**A**,**C**): control specimens; (**B**,**D**): exposed specimens; (**A**,**B**): white fibers; (**C**,**D**): red fibers. Insets: 1, 2, 3, 4: negative control of (**A**–**D**), respectively. The white fibers (wf) of control specimens (**A**) appear positive to CB1R, also after cocaine exposure (**B**), when they show an equal or slightly reduced positivity. The red fibers (rf) appear negative in the control specimens (**C**) and then become positive for CB1R after exposure to cocaine (**D**). The adipose tissue (at) appears always negative. Scale bar: (**A**–**D**): 0.050 mm. (**E**): semi-quantitative analysis of immunoreactivity. The Mann–Whitney test showed a statistically significant difference among the control and the exposed specimens (*p* < 0.05). White fibers of control specimens vs. white fibers of exposed specimens: * *p* = 0.0476; red fibers of control specimens vs. white fibers of exposed specimens: * *p* = 0.0238.

Immunohistochemical analysis showed a different expression of CB1R depending on the muscle fiber type. In the control specimens the white muscle fibers appeared immunopositive, while the red ones did not show positivity to CB1R (Figure 5). In cocaine-exposed specimens, white fibers showed a reduced immunopositivity, whereas the red fibers showed immunopositivity to CB1R (Figure 5). These results were confirmed by statistical analysis that showed statistically significant differences in the localization of CB1R among the control and the exposed specimens (*p* < 0.05) (Figure 5).

### 3.6. Skin and Gills Immunohistochemistry

**Figure 6:**
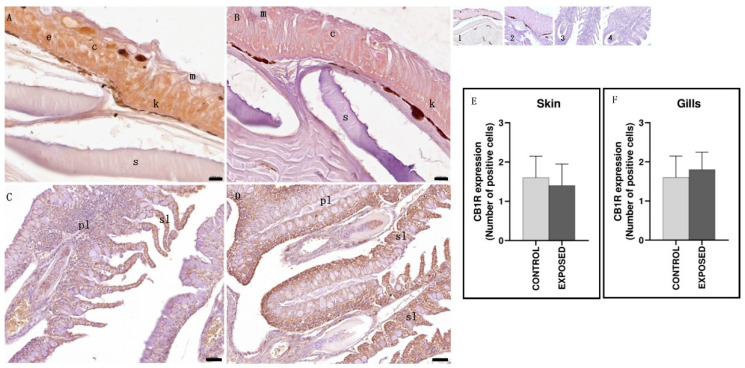
Light micrographs of eel skin and gills. Immunoreactivity to CB1R. (**A**,**C**): control specimens; (**B**,**D**): exposed specimens. Insets: 1, 2, 3, 4: negative control of (**A**–**D**), respectively. In the skin of control (**A**) and exposed (**B**) specimens, the epithelium (e) appeared immunopositive, with keratinocytes (k) and club (c) cells well stained. m: mucous cells; s: scales. The gills of both control (**C**) and exposed (**D**) specimens showed CB1R immunopositivity in the primary (pl) and secondary (sl) lamellae. Scale bar: (**A**,**B**): 0.020 mm; (**C**,**D**): 0.050 mm. (**E**,**F**): semi-quantitative analysis of skin and gills immunoreactivity, respectively. The Mann–Whitney test did not show a statistically significant difference among the control and the exposed specimens (*p* > 0.05).

As regards the skin, both in the control and the exposed specimens, CB1R positivity was observed in the epithelial layer, both in keratinocytes and club cells, but not in mucosal cells. No changes due to cocaine exposure were observed (Figure 6). Statistical analysis showed no statistically significant differences in the localization of CB1R between the control and the exposed specimens (Figure 6). As regards the gills, both in the control and the exposed specimens, a moderate immunopositivity to CB1R was observed both in primary and secondary lamellae. No changes due to cocaine exposure were observed (Figure 6). Statistical analysis showed no statistically significant differences in the localization of CB1R between the control and the exposed specimens (*p* < 0.05) (Figure 6).

## 4. Discussion

In our study, we used immunohistochemistry to collect evidence from the peripheral tissues of the European eel of the presence of CB1R, and its possible variations due to exposure to cocaine. As no eel-specific CB1R antibody was available, we used a rabbit antibody raised against the human CB1R. Indeed, the CB1R is well-conserved throughout evolution, as 97% of mouse CB1R, 84% of amphibian CB1R, and 72% of fish CB1R are identical to the human CB1 [44]. Moreover, we performed a Western blot analysis showing a molecular band of approximately 52 kDa, corresponding to the expected molecular weight of the CB1R protein. Therefore, we were confident that our antibody could recognize the eel protein.

Several conclusions can be drawn from the results of this study. First, the results of the immunohistochemical analysis showed for the first time the presence of the CB1R in the examined tissues of the European eel, although at different levels, as evidenced by the semiquantitative analysis. Secondly, the results showed that in many tissues, cocaine exposure was able to vary the expression of CB1R, suggesting a possible involvement of the endocannabinoid system in the mechanism of action of cocaine. Such involvement has been previously shown in the nervous tissue [31,32,33,34,35] but not in peripheral tissues.

The presence of CB1R immunopositivity was variable and varied in the different tissues examined. In the control specimens, immunopositivity appeared low/weak in the intestinal epithelium; moderate/strong in the renal hematopoietic tissue; evident in the degenerating and previtellogenic oocytes in the ovary and in the white fibers of striated muscle tissue; weak in the hepatic parenchyma; and moderate in the epidermis and gill epithelium. These results are consistent with data in the literature demonstrating the wide presence of CB1R in peripheral tissues in the animal kingdom [17,20,21,22,36,45,46,47] and in fish [17,18,30].

As regards the gut, in the intestinal epithelium and in the smooth muscle tissue of the control specimens, the presence of CB1R appeared to be very low, while the exposure to cocaine resulted in a significant increase in both tissues. Literature data indicate the presence of CB1R in the gastrointestinal system, where the receptor is abundant in the enteric nervous system, in non-neuronal cells such as intestinal mucosa and enterocytes, and in smooth muscle tissue [20,22,26,48]. Our results, which show a low presence of CB1R in the gut of the control specimens, contrast with those generally reported in the literature. There is no data on the presence of the CB1R in peripheral tissues of eel, or its expression in relation to different eel metamorphic stages, but one possible explanation could be that the specimens were in the silver stage, characterized by physiological bowel regression in preparation for reproductive migration, during which the eels do not feed [49]. CB1R is involved in the regulation of intestinal epithelium permeability, gastrointestinal motility, energy balance, and the increase in food intake, among others [22,40,50,51]; therefore, it is possible that in the silver stage, CB1R has been physiologically downregulated. Previous studies showed that the chronic exposure to cocaine increased the thickness of the epithelium and reactivated the structure of the intestine and of the intestinal musculature [52]. It could be assumed that the mechanism of action of cocaine involved the activation of the CB1R receptor, which is known to increase gastrointestinal epithelial integrity and tight-junctions proteins [53].

As for the liver, which is one of the main organs involved in the metabolization of cocaine [54], CB1R immunopositivity, which was poor in the control specimens, increased significantly after exposure to cocaine. Our results agree with those in the literature which report a low presence of the CB1R in the whole liver and hepatocytes, and its increase in various liver diseases, where it contributes to the underlying pathologies [55,56]. Such CB1R increase in human and mouse hepatocytes was observed under conditions of high fat or alcohol diets [57] and it was found to contribute to hemodynamic abnormalities and to promote hepatic lipogenesis [58] and fibrosis in liver cirrhosis [55]. Previous studies [59] found in the liver of eels chronically exposed to cocaine morphologic alterations such as necrotic areas and karyolitic and pyknotic nuclei, as well as increased cytochrome oxidase (COX) and caspase-3 activities, markers of oxidative metabolism and apoptosis activation, respectively. Moreover, an increase in serum levels of alanine aminotransferase (ALT), a marker of liver injury, was observed. The increase in CB1R in the liver of our cocaine-exposed eels agrees with the increase in CB1R observed in hepatic diseases. As far as further studies are needed, it could be assumed that the hepatotoxicity induced by cocaine involved the activation of the CB1R.

The teleost kidney is a mixed organ performing excretory and endocrine functions. It comprises hematopoietic, reticuloendothelial, endocrine, and excretory elements, as well as the nephrons, consisting of a glomerulus within Bowman’s capsule, proximal, distal, and collecting convolute tubules [60,61]. A moderate-to-strong CB1R immunopositivity was found in the hematopoietic tissue of the control specimens, whereas both the proximal and the distal tubules appeared generally immunonegative. After cocaine exposure, the immunopositivity of the hematopoietic tissue appeared absent/faint, whereas a mild-to-moderate immunopositivity to CB1R was observed only in the proximal tubules, causing a general decrease in kidney CB1R expression. Our results showing CB1R immunopositivity in the hematopoietic tissue of control specimens agree with those of studies that demonstrate the presence of CB1R in murine and human hematopoietic tissue, suggesting a role of these receptors in the regulation of immunity and blood cells development [52,62]. However, the strong attenuation of immunopositivity in the hematopoietic tissue of the exposed specimens needs further studies to be better evaluated. As for the presence of CB1R in the fish kidney, there is little literature; as far as we know, CB1R has been identified in goldfish [63] and zebrafish [46] kidney. Most studies were conducted on mammals, where CB1R was considered the predominant eCB kidney receptor. Its expression, low in healthy kidneys, has been identified in all components of the nephron and in the collecting duct; the activation of CB1R contributes to multiple renal diseases through the mitogen-activated protein kinase (MAPK) pathway [64,65,66]. Previous studies found, in the kidney of eels exposed to cocaine, nephrotoxic effects such as morphologic alterations and the increase in COX and caspase-3 activity, like that observed in the liver [58]. Our results showing immunonegativity in renal tubules of control eels and immunopositivity in proximal tubules of cocaine-exposed eels suggest an activation of CB1R induced by cocaine in proximal tubules. Interestingly, in the kidney, cocaine is metabolized to ecgonine methyl ester and norcocaine (NCOC), the latter being known to induce apoptosis in primary cultured human proximal tubular epithelial cells [67]. Therefore, although further studies are needed, it could be assumed that, at least partially, the nephrotoxicity induced by cocaine/norcocaine involved the activation of the CB1R, known to induce apoptosis in colon cancer cells [68].

Regarding the ovary, in the controls, CB1R was mainly expressed in previtellogenic and in mild vitellogenic oocytes. On the contrary, in cocaine-treated eels, CB1R immunopositivity was observed only in early developmental stages, with localization especially in previtellogenic oocytes. This distribution suggests that the early presence of the receptor may make immature follicles more vulnerable to the action of cocaine, thus contributing to the slowing of follicular progression. These observations are consistent with previous studies [69], in which cocaine-treated eels showed a reduced rate of ovarian maturation compared to controls. In these animals, in fact, there was a prevalence of previtellogenic oocytes, large areas of connective tissue, and larger and more numerous yolk vesicles. The increased expression of CB1R in control samples could therefore reflect the normal course of folliculogenesis, during which the receptor appears even in the most advanced stages, as indicated by the positivity observed in mid-stage vitellogenic oocytes. In cocaine-treated eels, on the other hand, follicular development slowed down by the action of the drug could determine the non-appearance of mid-vitellogenic oocytes; this phenomenon could therefore explain why CB1R levels in exposed people are lower than in controls. This is in line with the literature, where it is known that high levels of endocannabinoids and exogenous cannabinoids (abnormal activation of the endocannabinoid system) suppress the release of gonadotropin-releasing hormone (GnRH), luteinizing hormone (LH), follicle-stimulating hormone (FSH), estrogen, and progesterone. Furthermore, since mitochondrial function is associated with oocyte quality, the presence of CB1 on the outer mitochondrial membrane can disturb ovarian function and therefore oogenesis [70,71]. In control animals, the appearance of the receptor in mid-vitellogenic oocytes is in line with numerous instances of evidence in the literature confirming the key role of the endocannabinoid system in the regulation of folliculogenesis, oocyte maturation, and endocrine function of the ovary [72]. In mammals, CB1R has been identified in the ovaries, and an entire endocannabinoid system has been localized within human ovarian follicles, suggesting a regulatory function of these signals in follicular development [70]. Moreover, in mice, CB1R signaling reduces the ovulation rate and mRNA levels of some genes involved in ovulation [73]. In addition, CB1R has also been detected in the ovary of zebrafish [46], confirming the evolutionary conservation of this system in vertebrates.

As for the skeletal muscle, in the control specimens, the white muscle fibers appeared immunopositive and the red ones did not, whereas in cocaine-exposed specimens, white fibers reduced their immunopositivity, and the red fibers became immunopositive to CB1R. In humans and rodents, CB1R is localized on cell membranes and in mitochondria, and is involved in oxidative metabolism [74,75,76]. The observation of a prevalent association of the CB1 receptor with slow versus fast fibers suggests its role in influencing the type of muscle fiber [77]. The activation of CB1 is associated with metabolic dysfunction, chronic inflammation, and muscle loss, while its antagonism can improve insulin sensitivity, increase glucose absorption, reduce fat mass, and promote muscle growth through molecular pathways such as PI3K/Akt and mTOR [78]. Moreover, CB1R also regulates muscle apoptosis, and indeed agonism of CB1R induces various markers of apoptosis as caspase-3 in human embryonic rhabdomyosarcoma cells and in human primary and in rat cardiomyocytes [79]. Our results showing the presence of CB1R in the skeletal muscle of the European eel are consistent with literature data showing the presence of CB1R in skeletal muscle [74,75,76], but are in contrast with the prevalent association of CB1R with slow fibers; indeed, CB1R was associated with fast fibers in the control eels. The reason for this difference could be related to species differences and needs further studies to be evaluated. Previous studies showed in the skeletal muscle of cocaine-exposed eels signs of injury such as muscle breakdown and swelling, reminiscent of rhabdomyolysis, and these more evident in slow fibers; there was an increase in caspase-3 activity, and of serum levels of creatine kinase, lactate dehydrogenase, and aspartate aminotransferase, markers of the functional state of the muscle [80]. The decrease in the immunopositivity of fast fibers and the appearance of CB1R immunoreactivity in slow fibers after exposure to cocaine could be explained with a higher sensitivity of these fibers to cocaine, and with the greatest morphological damages observed in the slow fibers compared to fast ones. It is probable that the cocaine-induced injury involved the activation of CB1R in the slow fibers, where it was not present in normal conditions.

As for the skin and the gills, no changes due to cocaine exposure were observed in the CB1R immunopositivity that was found in the skin epithelial layer (keratinocytes and club cells) and in the gills primary and secondary lamellae. In humans and rodent skin, a functional eCB has been identified, involved in the modulation of cell growth, proliferation, and death. CB1R and CB2R immunoreactivities have been observed in human and murine skin cells, including epidermal keratinocytes, in which the activation of CB1R and CB2R by locally produced endocannabinoids induces suppression of cellular proliferation, differentiation, and the release of inflammatory mediators, as well as the induction of apoptosis [81,82]. As for the gills, as far as we know, CB1R was found in the gills of goldfish [63], whereas CB2a and CB2b receptors have been identified in the gills of zebrafish [83]. Our results, showing CB1R immunopositivity in the eel keratinocytes and in the gills, agree with those in the literature and suggest that probably even in the eel the eCB could exert a modulating function in these tissues; this, however, needs further studies to be evaluated. However, despite the morphological changes induced by cocaine in the epidermis (increased thickness of the epithelium and thickening and folding of the underlying basal lamina) [52] and in the gills (partial and total fusion of the secondary lamellae, proliferation of the interlamellar epithelium, epithelial lifting, and aneurism in the secondary lamellae) [84], there were no changes in CB1R immunopositivity in both tissues. Although further studies are needed, the action of cocaine could also involve receptors other than CB1R in these tissues.

## 5. Conclusions

In conclusion, the results of our study showed for the first time the presence of CB1R in some peripheral tissues of the eel, and changes in this receptor in many of these tissues after cocaine exposure, suggesting the involvement of CB1R in the response of the organism to cocaine. This involvement had already been observed in the nervous system, but not in peripheral tissues. In the different eel tissues, the alterations of the CB1 receptor were different, going from an increase to a decrease or a lack of alterations in the expression of the receptor; this difference could be explained by the fact that different signaling pathways were activated in different tissues, and/or even different receptors. The European eel is an endangered species; considering the important physiological activities carried out by the CB1R receptor in this, as in other, species, the study of the variations in this receptor, and of the endocannabinoid system more generally, can be of great use for the better knowledge and conservation of this species.

## Data Availability

The data supporting the conclusions of this article will be made available by the authors on request.
